# Mechanism of Electromechanical Coupling in Voltage-Gated Potassium Channels

**DOI:** 10.3389/fphar.2012.00166

**Published:** 2012-09-12

**Authors:** Rikard Blunck, Zarah Batulan

**Affiliations:** ^1^Groupe d’étude des protéines membranairesMontreal, QC, Canada; ^2^Department of Physiology, Université de MontréalMontreal, QC, Canada; ^3^Department of Physics, Université de MontréalMontreal, QC, Canada

**Keywords:** voltage-gated potassium channels, electromechanical coupling, gating, HCN, HERG, BK_Ca_

## Abstract

Voltage-gated ion channels play a central role in the generation of action potentials in the nervous system. They are selective for one type of ion – sodium, calcium, or potassium. Voltage-gated ion channels are composed of a central pore that allows ions to pass through the membrane and four peripheral voltage sensing domains that respond to changes in the membrane potential. Upon depolarization, voltage sensors in voltage-gated potassium channels (Kv) undergo conformational changes driven by positive charges in the S4 segment and aided by pairwise electrostatic interactions with the surrounding voltage sensor. Structure-function relations of Kv channels have been investigated in detail, and the resulting models on the movement of the voltage sensors now converge to a consensus; the S4 segment undergoes a combined movement of rotation, tilt, and vertical displacement in order to bring 3–4*e*^+^ each through the electric field focused in this region. Nevertheless, the mechanism by which the voltage sensor movement leads to pore opening, the electromechanical coupling, is still not fully understood. Thus, recently, electromechanical coupling in different Kv channels has been investigated with a multitude of techniques including electrophysiology, 3D crystal structures, fluorescence spectroscopy, and molecular dynamics simulations. Evidently, the S4–S5 linker, the covalent link between the voltage sensor and pore, plays a crucial role. The linker transfers the energy from the voltage sensor movement to the pore domain via an interaction with the S6 C-termini, which are pulled open during gating. In addition, other contact regions have been proposed. This review aims to provide (i) an in-depth comparison of the molecular mechanisms of electromechanical coupling in different Kv channels; (ii) insight as to how the voltage sensor and pore domain influence one another; and (iii) theoretical predictions on the movement of the cytosolic face of the Kv channels during gating.

## Introduction

Voltage-gated potassium channels (Kv) are a group of membrane proteins that regulate the flow of potassium ions into and out of cells in response to changes in the membrane potential. Kv channels are found throughout the body in different cell types. Their expression in neuronal and muscle tissues helps generate action potentials as well as maintain the resting membrane potential, thereby playing a critical role in cellular excitability in the central nervous and cardiac systems. Other roles of this class of proteins include regulation of hormone release such as the insulin secretion pathway (MacDonald and Wheeler, 2003) and implication in immune response (Koo et al., 1997; Beeton et al., 2001; Blunck et al., 2001; Thomas et al., 2011). Mutations in the genes encoding Kv channels lead to familial neuronal and cardiac diseases, including cardiac arrhythmias, episodic ataxia, epilepsy, and congenital deafness (Adelman et al., 1995; Neyroud et al., 1997; Jentsch, 2000; Tristani-Firouzi and Sanguinetti, 2003; Imbrici et al., 2006).

**Figure 1:**
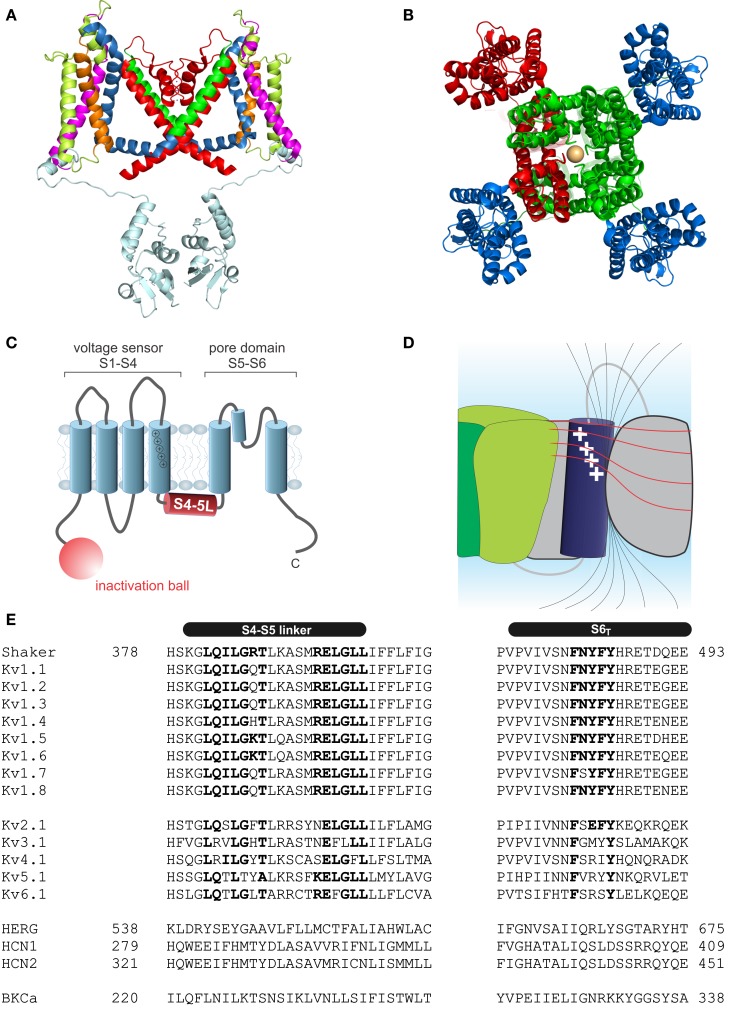
**Structure of voltage-gated potassium channels**. **(A)** *Side view* of the structure of Kv1.2/2.1 chimera (PDB: 2R9R, Long et al., 2007); in monomer A the segments S1–S6 are colored *magenta*, *lime*, *orange*, *blue*, *red*, and *green*, respectively. The T1 domain and the T1–S1 linker are shown in *light blue*. **(B)** Structure of Kv1.2/2.1 chimera (*top view*). One subunit is colored in *red*. For the other three voltage sensor domains are colored in *blue* and the pore domain in *green*. **(C)** Topology of Kv channels; S1–S4 form the voltage sensor, S5 and S6 together with the p-loop form the pore domain. The N-terminus contains the inactivation ball peptide. **(D)** The electric field inside the voltage sensor is concentrated on a few Ångström and is moving upon conformational change of the S4; *black* lines indicate the electric field and illustrate the concentration of the field, the *red* lines indicate equipotential planes (modified after Blunck et al., 2005; Chanda et al., 2005). **(E)** Sequence alignment of the S4–S5 linker and S6_T_ of different Kv channels (HERG alignment according to Ng et al., 2012). Accession numbers: *Shaker-CAA29917*; *Kv1.1-NP_000208*; *Kv1.2-NP_004965*; *Kv1.3-NP_002223*; *Kv1.4-NP_002224*; *Kv1.5-NP_002225*; *Kv1.6-NP_002226*; *Kv1.7-NP_114092*; *Kv1.8-Q16322*; *Kv2.1-NP_004966*; *Kv3.1-NP_004967*; *Kv4.1-NP_004970*; *Kv5.1-NP_002227*; *Kv6.1-NP_002228*; *HERG-BAA37096*; *HCN1-NP_066550*; *HCN2-EDL31671*; *BKCa-AF118141*.

Kv channels assemble as symmetric tetramers, with each subunit consisting of six transmembrane α-helices (S1–S6) connected by five linker regions. The first four helices (S1–S4) of one monomer form a distinct voltage sensor at the periphery, whereas the S5–S6 of all four monomers collectively arrange into a single ion conducting pore in the center of the structure (Figures 1A,B, Long et al., 2005a). Access to the ion conducting pore is controlled by an intracellular gate comprised of the S6 C-terminal ends, which form a bundle crossing that obstructs the pore when the channel is closed (Armstrong, 1971; Holmgren et al., 1997; Doyle et al., 1998). The S5–S6 linker forms a re-entrant loop (p-loop), arranging at the extracellular funnel into a small pore helix and the selectivity filter responsible for the preference for potassium over sodium in K^+^ channels (Doyle et al., 1998).

Each voltage sensor comprises a motif of four to six basic residues separated by two hydrophobic ones. This creates a positively charged surface along the S4 responsible for the sensitivity toward the membrane potential (Figure 1C). Driven by the positive charges in the S4 helix, the S4 transitions into the activated state (Liman et al., 1991; Papazian et al., 1991; Aggarwal and MacKinnon, 1996; Seoh et al., 1996). The S4 is partly accessible to the lipid environment, but the charged surface is directed toward the other helices of the voltage sensor S1–S3 (Long et al., 2005a). Voltage sensor and pore domain are covalently linked by the S4–S5 linker. The N-terminus forms a structure hanging below the ion channel pore (“hanging gondola,” Kreusch et al., 1998; Bixby et al., 1999; Long et al., 2005a) called the T1 domain (Figure 1A), which is responsible for the correct assembly of the tetrameric channels (Li et al., 1992; Shen and Pfaffinger, 1995).

## Kv Channel Gating

Upon depolarization of the membrane potential, the voltage sensors driven by the positively charged S4 undergo a conformational change (Mannuzzu et al., 1996; Cha and Bezanilla, 1997), which subsequently leads to pore opening. It has been shown that the S4 traverses several closed states before entering the activated state (Perozo et al., 1994; Zagotta et al., 1994b). Once all four voltage sensors are activated, the pore opens in one cooperative step (Zagotta et al., 1994a). The voltage sensor movement may electrically be detected as *gating currents* caused by the rearrangement of its electrostatic charges with respect to the electric field. The activation transitions are reflected in the gating currents as two major components – the first associated with the early closed-state transitions and the second with the major conformational change of the voltage sensor (Perozo et al., 1994). A fraction of the gating charge (∼13%) was also associated to the final concerted activation (Smith-Maxwell et al., 1998a; Ledwell and Aldrich, 1999; Pathak et al., 2005). Major charge movement was separated from the final cooperative transition and pore opening by three conservative mutations in the non-basic residues of the lower^1^ S4 (ILT mutation).

Combining the crystal structures of Kv1.2 and the Kv1.2/2.1 chimera (Long et al., 2005a, 2007) with a wealth of electrophysiological and voltage-clamp fluorometry results enabled to associate the kinetic transitions to structural features of the voltage sensor. One major landmark was the suggestion that the electric field does not homogenously drop off as it does within the membrane but that it reaches far into the voltage sensor along water-filled crevices from both faces in a manner that the field is concentrated onto a narrow span, a hydrophobic seal, between both sides (Figure 1D, Larsson et al., 1996; Starace and Bezanilla, 2001, 2004; Asamoah et al., 2003; Chanda et al., 2005; Tombola et al., 2005). Although a crystal structure is available only for the activated state, various models exist for the resting state and the gating movement of the voltage sensor. Starting from the sliding helix (Larsson et al., 1996; Yang et al., 1996) or helical screw model (Guy and Seetharamulu, 1986; Ahern and Horn, 2005), the transporter model (Starace and Bezanilla, 2001, 2004; Chanda et al., 2005), and the paddle model (Jiang et al., 2003; Ruta et al., 2005), the current understanding converges more and more toward a single consensus model for the gating movement of the voltage sensor (Khalili-Araghi et al., 2010; Vargas et al., 2011; Jensen et al., 2012; Yarov-Yarovoy et al., 2012). According to this consensus, the positive gating charges on the S4 are stabilized by pairwise interactions with anionic charges in S1–S3 aligned along the interface to S4 (Papazian et al., 1995; Tiwari-Woodruff et al., 2000; Yarov-Yarovoy et al., 2006). During activation, the positive charges “jump” from one negative charge to the following one leading to the conformational change of the voltage sensor. The movement of the S4 itself has been projected to be a combination of (i) a tilt of the S4 helix in the membrane, (ii) a rotation around the helix axis, and (iii) small vertical and radial translations. This movement will displace the S4–S5 linker and thus lead to pore opening (see below). In addition, it has been suggested that the S4 helix itself adopts a 3_10_ helical conformation permitting the helix to stretch and accommodate the continued stability of charged interactions (Long et al., 2007; Clayton et al., 2008; Villalba-Galea et al., 2008; Bjelkmar et al., 2009; Khalili-Araghi et al., 2010). The inner part of the S4 lengthens while the two ends twist around like a corkscrew. Whether the S4 adopts the 3_10_ conformation spontaneously or during activation is presently unknown. Upon prolonged stay in the activated state, S4 is then proposed to transform from a 3_10_ to an α-helix, which has been described as “relaxation” of the voltage sensor. (Villalba-Galea et al., 2008).

Pore opening itself is accomplished by a widening of the bundle crossing at the C-terminal S6. The S6 of many Kv channels contains a PVP motif leading to a kink of its axis (Figures 1A,E). It is assumed that, during pore opening, the S6 C-terminal to the PVP motif is moving away from the central axis thereby permitting entry into the central water-filled cavity. Opening of the pore, however, triggers inactivation of the channel. Two major types of inactivation have been described, N- and C-type inactivation. During the fast, N-type inactivation, a ball peptide tethered to the N-terminus of the Kv channels enters the open pore and blocks access to it (Armstrong and Bezanilla, 1977; Hoshi et al., 1990; Zagotta et al., 1990). During slow, C-type inactivation, the selectivity filter acts as a second gate and prevents ions from passing through (Yellen, 2002; Blunck et al., 2006; Cordero-Morales et al., 2006a,b). Opening of the lower gate directly triggers the slow entry into the C-type inactivated state (Cuello et al., 2010b,c), implying that the two gates of the ion conducting pore act diametrically – opening of the cytosolic gate triggers closing of the extracellular one.

## Electromechanical Coupling

As described above, the energy driving the opening of the pore is generated by the voltage sensor upon changes of the surrounding *electric field*. Accordingly, *electromechanical* coupling describes the process of transferring this energy from the voltage sensor to the pore domain, triggering the *mechanical* opening of the pore. By first approximation, the voltage sensor movement pulls the lower S5 helix outward via the only covalent link, the S4–S5 linker. However, a number of questions remain unanswered by this simplified view. First, the major voltage sensor movement seems to occur independently followed by a single cooperative step that is associated with pore opening (Zagotta et al., 1994a; Pathak et al., 2005); in other words, all four voltage sensors have to be activated before the final pore opening step is allowed. The major (charge) movement of the voltage sensor thus has to happen independently of pore domain opening, arguing against a direct coupling between both movements. Second, it is not clear how the conformational rearrangement of the S4 mechanically leads to a widening of the helical bundle crossing. Third and finally, while the S5 is covalently linked to the voltage sensor, it is the S6 that obstructs the ion conduction pathway. How are both helices linked to one another? Some of these questions have already been answered to date; others remain the focus of research. Below, we will outline the results that led to the current understanding of the mechanisms of electromechanical coupling and discuss the open problems. Initially, we will concentrate on the Shaker-like Kv channels as the fundamental model system and compare the mechanism with results obtained in other voltage-dependent potassium channels.

## Electromechanical Coupling in Shaker-Like Kv Channels

Early on, it was found that the covalent link between voltage sensor and pore, the S4–S5 linker plays a key role in the electromechanical coupling. Slesinger et al. (1993) already identified positions in the S4–S5 linker that influence the properties of the permeation pore, but at that time, the linker was still assumed to be part of the ion conducting pathway. Its involvement in the intermediate (coupling) transitions was first proposed by Schoppa and Sigworth (1998a,b) and, for HERG channels (Human ether-a-go-go related gene, see below), by Sanguinetti and Xu (1999). However, as it is the S6 – not the S5 helix that lines the pore (Liu et al., 1997; Doyle et al., 1998), it remained unanswered as to how movement of the S4–S5 linker led to pore opening itself. Lu et al. (2001, 2002) solved this problem by demonstrating that the S4–S5 linker directly interacts with the C-terminal S6 (S6_T_) promoting pore opening. They constructed Shaker-KcsA chimeras by replacing the Shaker pore with the corresponding KcsA domain. These constructs were gating voltage dependently only if the corresponding S4–S5 linker and S6_T_ were paired. The involvement of S6_T_ in electromechanical coupling was corroborated by mutations in this region leading to altered coupling (Ding and Horn, 2002, 2003; Hackos et al., 2002; Soler-Llavina et al., 2006; Labro et al., 2008; Batulan et al., 2010; Haddad and Blunck, 2011). Lu et al. (2002) showed that both the motifs _483_YFYH_486_ in the S6_T_ and _385_LGRTLKAS_392_ in the S4–S5 linker were essential, although these regions should probably be extended to _481_FNYFY_485_ and _382_LQILGRT_388_ (Figure 1E, McCormack et al., 1991; Schoppa and Sigworth, 1998a; Soler-Llavina et al., 2006; Labro et al., 2008; Haddad and Blunck, 2011). In the crystal structure of Kv1.2, these are also the regions that make the closest contact between both regions of the same subunit (Long et al., 2005b, Figure 2A). It is suggested that F481, Y483, and F484 form a hydrophobic pocket, into which the S4–S5 linker and in particular I384 and T388 insert (Labro et al., 2008; Haddad and Blunck, 2011). Sequence alignment of the S4–S5 linker and S6_T_ regions shows that the motifs are conserved among Kv1 family members. In Kv2-6, few variations occur with L382, L385 in the S4–S5 linker and F481, Y485 in the S6_T_ being strictly conserved (Figure 1E).

**Figure 2:**
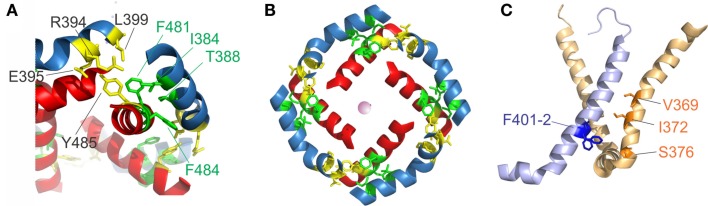
**S4–S5 linker and S6_T_ interaction**. **(A)** Region of annealing between the S4–S5 linker (*blue*) and the S6_T_ (*red*). Residues identified in intrasubunit interaction (I381, T388, F481, and F484) are colored in *green*, residues involved in intersubunit interactions (R394, E395, L399, and Y485) are colored in *yellow*. **(B)** Coordination of the S4–S5 linker and the S6_T_ in the crystal structure. The S4–S5 linkers form a ring and enclose the S6_T_ between two S4–S5 linkers. [coloring as in **(A)**]. **(C)** Residues involved in direct contact of S4 (*light orange*) with the neighboring S5 (*light blue*) are shown in *blue* (F401, F402) and *orange* (ILT: V369, I372, S376).

Lu et al. (2002) also found the C-terminal part of the S4–S5 linker (_393_MRELGLL_399_) to be essential although it does not make direct contact in the open state structure. However, it had been demonstrated that this region interacts with Y485 (in the _483_YFYH_486_ motif) of the neighboring subunit (Batulan et al., 2010). Three of the residues involved, E395, L399, and Y485 are strictly conserved throughout Kv1-6 (Figure 1E). Mutations in this region influence primarily the deactivation of off-gating kinetics, indicating that this interaction develops in the open state only. When looking at the three-dimensional arrangement of all four S4–S5 linker and the S6_T_ (Figure 2B, Long et al., 2007), it becomes evident that the _481_FNYFYH_486_ motif of the S6_T_ is nestled between the N-terminal part of the S4–S5 linker of the same subunit (_382_LQILGRT_388_) and the C-terminal part of the neighboring S4–S5 linker (_394_RELGL_398_). The formation of an intersubunit link between the S4 and S5 linker of one subunit with the S6_T_ of the neighboring one (Batulan et al., 2010) has also very recently been proposed to develop after pore opening in the prokaryotic sodium channel Nav-Ab (Payandeh et al., 2012). In Nav-Ab, the interaction is located more toward the center of the S4–S5 linker possibly because Nav-Ab undergoes a different type of inactivation (see below).

### Alternative interaction regions

Despite the fact that the most profound interactions on the electromechanical coupling were found in the S4–S5 linker and S6_T_ region, other contacts between the voltage sensors and the pore domain seem to influence electromechanical coupling as well. These may explain why still a voltage-dependent opening of the pores is observed in “uncoupled” mutants (Haddad and Blunck, 2011)^2^. Mutations in the region of N-terminal S5 (401–405) and the S6 in the region of the PVP kink (472–479) have a similar, although less pronounced, effect as uncoupling mutations (Kanevsky and Aldrich, 1999; Soler-Llavina et al., 2006). According to the crystal structure of the Kv1.2/2.1 chimera (Long et al., 2007), these positions are oriented toward the voltage sensor of the neighboring subunit, right at the area where the ILT mutations (V369I-I372L-S376T) are located (Figure 2C). Soler-Llavina et al. (2006) suggested therefore a direct annealing between the lower S4 and the S5 of the neighboring subunit. This is different from the suggestion of Batulan et al. (2010) of an intersubunit interaction between the S4–S5 linker and the S6 of the neighboring subunit. As the residues involved in the interaction between the S4 and the neighboring S5 are all hydrophobic, an influence of membrane lipids positioned at the interface of the voltage sensor and pore domain is also possible (Soler-Llavina et al., 2006).

The lipid composition had been shown to influence activation of the prokaryotic KvAP (Schmidt et al., 2006, 2009) and Shaker K^+^ channels (Borjesson et al., 2008, 2010; Xu et al., 2008). In the bacterial voltage-gated KvAP channel, it has been proposed that the positively charged arginine residues along the voltage sensor interact with and are stabilized by negatively charged lipid phosphodiester groups (Schmidt et al., 2006). By changing the lipid environment from a phospholipid to a non-phospholipid make-up, the voltage sensor switches from an activated to a resting state. Similarly, enzymatic cleavage of the phospholipid head groups hinders Shaker K^+^ activation (Ramu et al., 2006; Xu et al., 2008). Recently even binding sites for polyunsaturated fatty acids had been identified (Decher et al., 2010; Borjesson and Elinder, 2011). Nevertheless, these interactions seem to be electrostatic in nature and do not seem to target the coupling between voltage sensor and pore domain.

Two other contact regions have been implicated in electromechanical coupling. First, the crystal structure predicts the upper S5 to be in close contact with the upper S4 of the neighboring subunit. Close proximity of these regions had been proposed earlier (Elinder et al., 2001a,b; Laine et al., 2003) although mutations in this region did not seem to energetically uncouple voltage sensor and pore. The interaction may therefore play a minor functional role, or have effects primarily on gating kinetics (Soler-Llavina et al., 2006).

The second contact area involves S1 and the pore helix (Lee et al., 2009). This was identified, in addition to the S4–S5 linker, based on a statistical coevolution analysis of Kv channels. Crosslinking of S1 to the pore helix in the prokaryotic KvAP channels prevented channel opening to a certain extent. The authors suggest that a S1-pore helix interaction acts as an anchor to facilitate coupling via the S4–S5 linker.

### Energetic considerations in electromechanical coupling – elasticity

**Figure 3:**
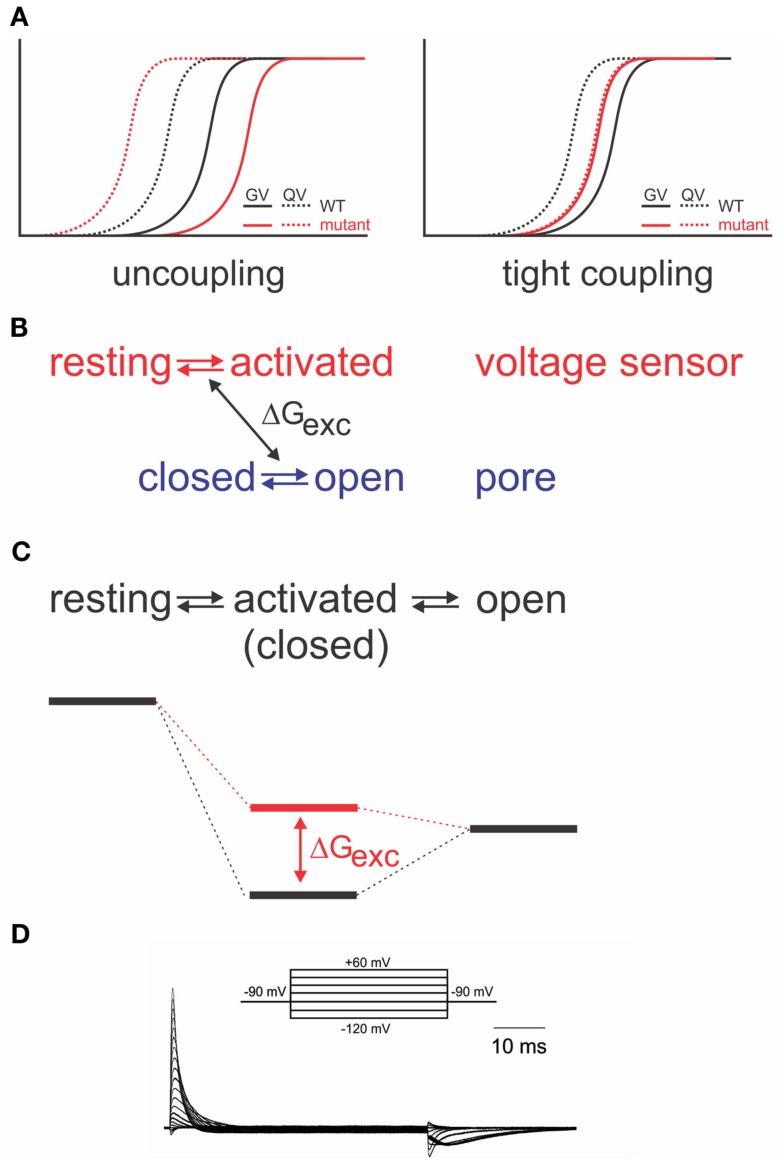
**Energetic coupling between voltage sensor and pore domain**. **(A)** Change of gating charge–voltage (*QV*) and conductance–voltage (*GV*) relations for uncoupled (*left*) and tightly coupled (*right*) mutants (modified from Haddad and Blunck, 2011). **(B)** Relation between two separate models and exchange of free energy between voltage sensor and pore domain. **(C)** Analogous sequential model and associated energy diagram. Change of coupling energy Δ*G* alters energy level of activated state. Charges are moved during resting-activated transition. **(D)** Gating currents elicited from the Shaker-W434F mutant in response to pulses from −90 to potentials varying between −120 to +60 mV in 10 mV intervals.

As it is required for all four voltage sensors to have moved before the pore opens in a single cooperative step (Zagotta et al., 1994a), the system has to contain a certain amount of “elasticity,” where the energy provided by the activation of a single voltage sensor is “stored” until all four voltage sensors have been activated. The structural basis for the elasticity remains unknown; it shows, however, in the energetics of the electromechanical coupling. Uncoupling by point mutations (Ledwell and Aldrich, 1999; Ding and Horn, 2003; Soler-Llavina et al., 2006; Haddad and Blunck, 2011) separates gating charge–voltage (*QV*, reflecting voltage sensor movement) and conductance–voltage (*GV*) relations (reflecting pore opening); while the *QV* is shifted to more negative potentials, the *GV* is shifted to more positive ones (Figure 3A). This is a distinct property of disturbed electromechanical coupling, as stabilizing or destabilizing either the voltage sensor in its resting state or the pore in its open state would lead to symmetric effects on *QV* and *GV* (Ding and Horn, 2003; Batulan et al., 2009; Muroi et al., 2009, 2010; Haddad and Blunck, 2011). The shift of the *QV* to more negative potentials means that less energy is required to activate the voltage sensors. In the wildtype channel, this energy is likely transferred to the pore, indicating that the pore itself prefers to remain in the closed state. A separation between *QV* and *GV* can only be achieved by modifying the energetic coupling between both modules (Batulan et al., 2009; Muroi et al., 2009, 2010; Haddad and Blunck, 2011).

If the system is considered as two entities, voltage sensor and pore, which can transit from resting to activated and closed to open, respectively, then a certain amount of energy (Δ*G*_exc_) is exchanged between both systems (Figure 3B). Muroi et al. (2010) modeled the behavior of the two systems as cooperatively coupled. The cooperative models imply that the pore has a relatively high probability of opening in the voltage range where the voltage sensors become activated^3^. Uncoupled mutants, however, seem to have a very low open probability in that region (Ledwell and Aldrich, 1999; Soler-Llavina et al., 2006; Muroi et al., 2010; Haddad and Blunck, 2011). Considering, furthermore, the tight interaction between voltage sensor and pore discussed above, it is likely that their respective free energies are largely dependent on the presence of the other module. Thus, it is more prudent to describe the energetic states of the *entire* system in a sequential model (resting-activated-open; Figure 3C; Haddad and Blunck, 2011), where the charge *Q* is moved during the closed-activated transition. In this description, the energy exchanged between voltage sensor and pore domain will influence the energy level of the intermediate, “activated” state. As the energy required to open the pore has to be available prior to entering the activated state, the activated state becomes higher energetic (Figure 3C). It is important to note that the final opening transition in the models is inevitably voltage dependent in order to explain the voltage dependence of the “uncoupled” mutants’ *GV* (Ledwell and Aldrich, 1999; Soler-Llavina et al., 2006; Haddad and Blunck, 2011). The default state of the pore in different Kv channels has in detail been discussed in Vardanyan and Pongs (2012, in this topic issue).

Interestingly, the separation of *QV* and *GV* can be inversed by a single point mutation (I384A) in the S4–S5 linker of the Shaker channel (Haddad and Blunck, 2011). In the I384A mutant, *QV* and *GV* superpose, indicating that voltage sensor movement is tightly coupled to pore opening (Figure 3A). In the energetic model, the transition from the activated to the open state occurs immediately. Physically, it means that a single subunit is no longer “permitted” to enter the activated state without opening the pore. It is likely that due to the tight coupling, more energy is transferred to the pore enabling it to open at lower potentials (Haddad and Blunck, 2011).

### Spring or bolt?

This leads us to the question about the nature of the link between the voltage sensor and pore. What can we say about the movement of S4–S5 linker and S6_T_ relative to one another? In principle, two scenarios are possible. The first possible scenario is that the S4–S5 linker and S6_T_ remain in close contact during activation. The S4 and/or the S4–S5 linker act as a spring that becomes strained or compressed by the activation of the voltage sensors. If sufficient energy is stored, the S6_T_ is pushed or pulled open. In the second scenario, the S4–S5 linker acts as a bolt that prevents the pore from opening. Only once all four bolts are removed, i.e., the S4–S5 linkers have moved out of the way, the pore passively follows the opening. In the second scenario, the energy for opening of the pore is not provided by the voltage sensor; instead the voltage sensors “break” the interaction between S4–S5 linker and S6_T_. As a consequence, only the *QV*, not the *GV* should be affected by uncoupling.

In both scenarios the interaction between S4–S5 linker and S6_T_ has to occur in the closed state. An interaction that occurs in the open state would lead to an *increase* in the energy of the activated state and shift the voltage dependence (*QV*) to more *positive* potentials when disturbed, contrary to the experimental observations. The interaction has to occur thus in the closed state. The same conclusion has been found based on disulfide bridges in HERG channels (Ferrer et al., 2006) and by interaction with complementary peptides in KCNQ1 channels (Choveau et al., 2011), both of which are thought to have similar coupling mechanisms (see below, Labro et al., 2011).

The same two possibilities exist for channel deactivation as for activation; the S4–S5 linkers could return to the deactivated state leaving the S6_T_ to follow passively or the S6_T_ could be pulled or pushed back into its resting position by the S4–S5 linker. The question remains, therefore, whether the N-terminus of the S4–S5 linker keeps in close contact with the S6_T_ throughout the gating process. Several results indicate that both regions are rather “loosely” coupled, as first suggested for HCN channels by Chen et al. (2001). First, the strength of the coupling varies considerably in response to small changes, e.g., I384N and I384A in Shaker lead to uncoupling and tight coupling, respectively. If the link would remain intact continuously, these effects should not be observed. A sliding or detaching between both helices is thus more likely. Accordingly, the HERG channel can be locked in the closed state by a disulfide bridge between S4–S5 linker and the S6_T_ (Ferrer et al., 2006). Second, Choveau et al. (2011) showed that co-expressed peptides of the respective complementary helix influenced gating. Nevertheless, it cannot be excluded that this already occurred during protein folding. Third, in HCN channels (Chen et al., 2001) as in the Kv-KcsA chimera (Lu et al., 2001, 2002), disturbance of the coupling by mismatch of S4–S5 linker and S6_T_ led to a fraction of the channels being constitutively open. In contrast, uncoupling in Shaker led to constitutively closed channels (Smith-Maxwell et al., 1998b; Ledwell and Aldrich, 1999; Soler-Llavina et al., 2006; Haddad and Blunck, 2011). The difference is likely found in the “preferred” state of the pore itself. KcsA, gated by protons (Heginbotham et al., 1999), is held closed by a series of pH-dependent interactions at the helical bundle crossing (Takeuchi et al., 2007; Thompson et al., 2008; Cuello et al., 2010a). Upon increase in proton concentration, release of the interactions and electrostatic repulsion of the charged residues leads to pore opening. As the transition to the open state occurs spontaneously devoid of additional energy sources, the pore opens by itself and the Kv-KcsA chimera need to push the pore closed. The negative shift of the *QV* in the uncoupled Shaker mutants, on the other hand, demonstrates that energy is required to bring the channel into the open state. In addition, no voltage independent component is observed in Shaker channels even at open probabilities as low as 10^−6^ (Islas and Sigworth, 1999). Thus, although the pore is not locked in the closed state, it will not open spontaneously and needs to be pushed (or pulled) open. The structural basis for the inherently different behavior of the pores might be based on the PVP motif found in many Kv channels but not in HCN, HERG, or KcsA (Figure 1E). This is supported by the fact that mutation of the second proline in the PVP motif to an aspartate leads to a constitutively open channel with only fractional voltage dependence (Sukhareva et al., 2003). This must be seen in the context of the general pore architecture and cannot be generalized. As we will discuss below, the HCN channels’ default state is closed despite the absence of a PVP motif. The other way around, HERG channels become constitutively open by introducing a PVP motif in the S6 (Fernandez et al., 2004; for a detailed discussion of constitutive conductance see also Vardanyan and Pongs, 2012).

Finally, in the tightly coupled mutant I384A, the voltage sensors are held back in their activated state during deactivation leading to very slow closing kinetics (Haddad and Blunck, 2011). All of the above suggest that during deactivation, the voltage sensors “separate” from the S6_T_, and that the pore follows passively. While the Kv-KcsA chimeras need to be pushed closed (Lu et al., 2002), wildtype Shaker channels need to be pushed open (Haddad and Blunck, 2011).

### Stabilization of the activated state

Just like the annealing of the N-terminus of the S4–S5 linker to S6_T_, also the interaction between the lower S4 and S5 of the neighboring subunit seem to follow a similar pattern and develop in the closed state (Smith-Maxwell et al., 1998a,b; Ledwell and Aldrich, 1999; Pathak et al., 2005; Soler-Llavina et al., 2006). Other influences on the voltage sensor movement, on the other hand, develop mainly in the open state. The gating currents of the non-conducting mutant W434F (Perozo et al., 1993; Bezanilla et al., 1994; Stefani et al., 1994) are asymmetric for the beginning and end of a depolarizing pulse (Figure 3D). The slow rising phase of the off-gating currents was attributed to an interaction developing between the C-terminus of the S4–S5 linker and the S6_T_ of the neighboring subunit (Batulan et al., 2010). The interaction stabilizes the pore in the open position during the final transitions of activation. The pore thus “pushes” less onto the voltage sensors so that they require more energy to return to their resting state.

This effect is also one of the reasons for the shift of the voltage dependence during prolonged depolarizations (Fedida et al., 1996; Olcese et al., 1997, 2001; Haddad and Blunck, 2011; Lacroix et al., 2011). The voltage sensor no longer feels the pore pushing, so that it follows a voltage dependence shifted to more negative potentials. However, the structural implications are not restricted to the pore region but also lead to conformational changes in the voltage sensor (Bruening-Wright and Larsson, 2007; Villalba-Galea et al., 2008; Haddad and Blunck, 2011). The conformational change or “relaxation” of the voltage sensor has not been observed in uncoupled Shaker mutants (Gagnon and Bezanilla, 2010; Haddad and Blunck, 2011), and the related slowing of deactivation kinetics are not observed if pore opening is blocked (Batulan et al., 2010; Lacroix et al., 2011). Thus, through the electromechanical coupling, a conformational change is allosterically induced in the voltage sensor domain. On the other hand, the conformational changes are observed in the isolated voltage sensor of the voltage-gated phosphatase CiVSP (Villalba-Galea et al., 2008).

It is thought that the S4 adopts a 3_10_ helical structure in resting state or during activation in order to better pair the positive charges with the negatively charged counterparts in the S1–S3 and relaxes in the activated state to an α-helical structure (Clayton et al., 2008; Villalba-Galea et al., 2008; Catterall, 2010; Chakrapani et al., 2010). This is a mechanism similar to open state stabilization occurring in the pore described above (Batulan et al., 2010). In both cases, the channel when entering the activated open state is not immediately in its optimal coordination. The side chains have to reorient themselves and adapt to their new environment, and during this adjustment new links form stabilizing the open state of both pore and voltage sensor.

## Model for Movement of the S4–S5 Linker

Presently, only a crystal structure for the presumably open inactivated state of Kv channels is known (Long et al., 2005a, 2007). Several models have been proposed on the closed state (see above), which are constrained by biophysical data obtained from the extracellular face of the channel. The movement of the lower S4 and the S4–S5 linker is currently extrapolated in molecular dynamics simulations (Khalili-Araghi et al., 2010; Vargas et al., 2011; Jensen et al., 2012; Yarov-Yarovoy et al., 2012). Based on the open state crystal structure and the results presented above, one can make predictions about the mode of action of the S4–S5 linker and its interaction with the surrounding environment. Figure 2B shows the relative orientation and the interactions relevant to electromechanical coupling in the Kv1.2/2.1 chimera (Long et al., 2007). Starting from this conformation, the S4 will translate to a certain extent downwards combined with a tilt and rotation and will pull the S4–S5 linker with it. The slow component in the gating indicates that the intersubunit interaction between the S4–S5 linker and the neighboring S6T (Figures 2A,B, yellow, Batulan et al., 2010; Payandeh et al., 2012) will break during this movement and leave the S6_T_ to follow the S4–S5 linker. Although the coupling between S6_T_ and S4–S5 linker exists in the open and closed state, the “elastic” nature of it allows for a relative sliding or temporary separation between both helices. During closing the S6 will be straightened and the S4–S5 linker will push the S6_T_ inwards as suggested by Yarov-Yarovoy et al. (2012). The movement will tilt the N-terminus of the S4–S5 linker inwards, which would mean that it would move close to the S5. In order to keep the S4–S5 interaction (Figure 2C, Smith-Maxwell et al., 1998b; Ledwell and Aldrich, 1999; Soler-Llavina et al., 2006) intact also in the closed state while achieving a horizontal tilt in the S4–S5 linker, both S4 and S5 have to rotate during closure. Similar movements had been suggested based on molecular dynamics simulations. However, in order to be confident about the movement of the internal face of the channel during gating, either a closed-state crystal structure or dynamic structural data as recently presented (Faure et al., 2012) are required.

## Relation to Other Voltage-Gated Potassium Channels

The above discussion evolved mainly around the electromechanical coupling of the Shaker Kv channels. Some questions remain however – first, in how far the mechanisms found in these channels are conserved in other voltage-gated potassium channels; second, how are they modulated in order to accommodate hyperpolarization-activated channels; third and finally, how does the voltage sensor machinery interact with other activators such as Ca^2+^ or cyclic nucleotides.

### Delayed rectifier Kv channels

**Figure 4:**
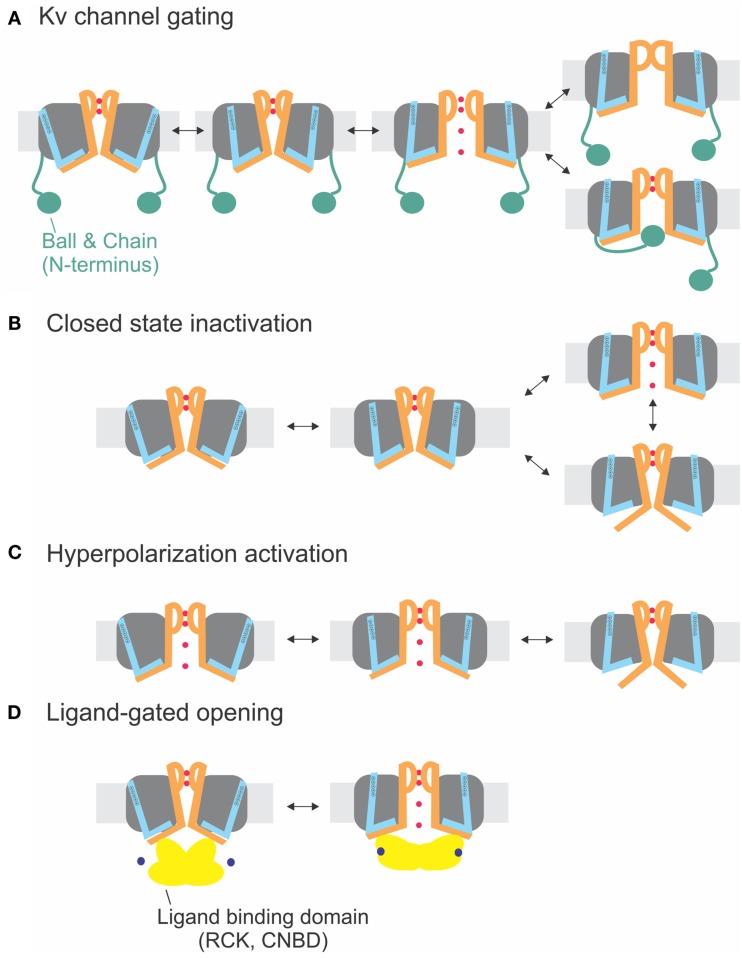
**Electromechanical coupling types**. **(A)** Electromechanical coupling in Shaker channels. Activation of the S4 (*blue*) applies strain onto the link to the S6_T_ (*S6 orange*), and finally leads to pore opening. Inactivation occurs by N-type inactivation (ball and chain; *bottom*) or C-type inactivation (selectivity filter; *top*). **(B)** Closed-state inactivation: Closed-state inactivation (*bottom*) is caused by a loose coupling between S4–S5 linker and S6_T_; upon decoupling, the pore enters its default state (closed). **(C)** Coupling in hyperpolarization-activated channels: Activation of the S4 leads to decoupling between S4–S5 linker and S6_T_ and closing of the pore. **(D)** Ligand-gated opening. The ligand binding domains are linked to the S6_T_ directly and apply their energy directly to the S6.

The sequences of the S4–S5 linker and the S6_T_ (Figure 1E) do not significantly vary between the different delayed rectifier potassium channels. Accordingly, the close interaction between both regions has been demonstrated for several members of these families such as Kv1.5 (KCNA5, Labro et al., 2008), Kv2 (KCNB, Jara-Oseguera et al., 2011), Kv4 (KCND, Bhattacharji et al., 2006; Barghaan and Bahring, 2009), KvLQT (KCNQ, Choveau et al., 2011; Labro et al., 2011), and HERG (KCNH, see below, Sanguinetti and Xu, 1999; Tristani-Firouzi et al., 2002; Ferrer et al., 2006; Van Slyke et al., 2010). In contrast to Shaker-related Kv channels, Kv4 channels enter an inactivated state directly from the closed state at low potentials (Jerng and Covarrubias, 1997; Bahring et al., 2001; Bähring and Covarrubias, 2011). This so-called *closed-state inactivation* is distinct from both N- and C-type inactivation in Shaker-related Kv channels. It has been suggested by Barghaan and Bahring (2009) to be caused by the loose interaction between the S4–S5 linker and the S6_T_. The S6_T_ separates from the S4–S5 linker during opening or in the open state leading to reclosure of the pore. In this case, the pore’s default state would be closed as in the other Kv channels containing a PVP motif. Accordingly, inactivation is prevented by the mutation of the PVP motif to PVA (Bhattacharji et al., 2006). The loss in contact between S4–S5 linker and S6T during inactivation has also been suggested for HCN channels (see below) and would be in accordance with the crystal structure of the inactivated state of NaV-Ab (Payandeh et al., 2012). It is therefore likely that Kv4 and NaV-Ab undergo the same type of inactivation where the cytosolic pore gate closes due to loss of contact to the S6_T_ (Figure 4B).

### Hyperpolarization-activated cyclic nucleotide-gated channels

Hyperpolarization-activated cyclic nucleotide-gated (HCN) channels are expressed in pacemaker cells found in the heart and the nervous system. HCN channels are activated at hyperpolarized, rather than depolarized, potentials, and are selectively conducting cationic inward currents known as *I*_f_ in the heart (DiFrancesco, 1993) and *I*_h_ in neurons (Pape et al., 1996). These currents contribute to the slow depolarization of pacemaker cells, which is important for changes in heart rate and maintenance of neural oscillatory networks (Santoro and Tibbs, 1999). Although this group shares significant sequence homology and channel architecture with Kv channels (Santoro et al., 1998; Shin et al., 2001; Rothberg et al., 2002), they differ in that they contain a cytoplasmic cyclic nucleotide binding domain (CNBD) attached to the S6 C-terminus. Binding of cyclic nucleotide, primarily cyclic AMP, to this domain shifts HCN channel activation to more positive potentials (Gauss et al., 1998; Shin et al., 2001; Wainger et al., 2001).

Despite the absence of any similarity to the S4–S5 linker and the S6_T_ region of Kv channels, HCN channel activation appears coupled to the movement of the voltage sensor via annealing of the S4–S5 linker to S6_T_. Alanine-scanning mutagenesis of the S4–S5 linker identified three residues (E324, Y331, and R339)^4^ that may be important in channel closing (Chen et al., 2001). Various mutations in these positions increased the minimal open probability when expressed in oocytes, thus channels stay open even at very large depolarizations. Later results suggested that one of these S4–S5 linker amino acids, R339, interacts with a residue on the C-terminal end of the S6, D443, forming a salt bridge, and that this interaction may be modified by a nearby residue, R447 (Decher et al., 2004). The findings indicated that, similar to Kv channels, electromechanical coupling in HCN channels is mediated from the S4–S5 linker to the S6_T_. However, despite the similarities in the coupling mechanism, two properties of HCN channels distinguish them from Kv channels. First, the channel is closed rather than opened by the movement of the S4–S5 linker in response to depolarization. Second, the S6_T_ is the covalent link to the CNBD, which means that both interaction partners, S4–S5 linker and S6_T_, are linked to an activating module, the voltage sensor and the CNBD, respectively. S6_T_ and the following C-linker enable the gating by cyclic nucleotides in cyclic nucleotide-gated channels (Wang et al., 2001; Zagotta et al., 2003).

Although the channel opens at hyperpolarized potentials, the voltage sensor movement itself shows all the characteristics of Shaker channels (Mannikko et al., 2002; Bruening-Wright et al., 2007). The voltage dependence of opening can even be reversed to open at depolarizing potentials by cross-bridging the distal C-linker with the S4–S5 linker (Prole and Yellen, 2006). Yellen and co-workers (Shin et al., 2001, 2004; Rothberg et al., 2002) therefore proposed that during “desensitization” of the channel, i.e., closing at depolarized potentials, the coupling between the voltage sensor and the pore “slips” or gets separated. Therefore, no energy is transferred any longer to the pore, and the pore closes. Despite its similarity, the process is different than the closed-state inactivation proposed for Kv4 channels (see above and Barghaan and Bahring, 2009; Bähring and Covarrubias, 2011; Payandeh et al., 2012). Kv4 channels first open and then inactivate with a temporal delay whereas the HCN channels are open and only close in response to depolarization. In both cases, the pore’s default state is closed, and the voltage sensor brings it into the open state. In HCN channels, however, the resting state is already open, and the gating movement of the S4–S5 linker tries to open it even further, which leads to separation and closing of the pore (Figures 4B,C).

The second feature distinguishing HCN channels from most other Kv channels, the cytosolic CNBD, is linked to S6_T_ via the C-linker. From the crystal structures of the C-linker and CNBD of several CNG channels (Zagotta et al., 2003; Xu et al., 2010; Lolicato et al., 2011; Brelidze et al., 2012), it is known that the CNBDs form a tetrameric ring below the transmembrane pore that interacts via the C-linker onto the S4–S5 linker. This indicates that both voltage and cAMP-binding act upon the same gate (Shin et al., 2004), suggesting that cAMP-binding modulates the coupling efficiency between S4–S5 linker and S6_T_. Binding of cAMP to HCN2 channels leads to a shift of channel closing to more depolarized potentials. Also cleaving the CNBD leads to a shift to more depolarized potentials (Wainger et al., 2001), indicating that the CNBD facilitates hyperpolarization-activated closing of the channel and that cAMP binding inhibited the closing (Wainger et al., 2001; Craven and Zagotta, 2006). Interpreting this in view of a separation between S4–S5 linker and S6_T_, on the other hand, the separation occurs at higher potentials indicating that the strain between both modules is relieved by cAMP binding. Although the exact mechanism is not known, cAMP binding stabilizes the pore in its open position perhaps by “pulling” the S6 termini outwards (Figure 4D). Stabilization of the open pore would also explain the higher current induced by cAMP in HCN2 channels (Wainger et al., 2001).

### HERG channels

Human ether-a-go-go related gene (HERG, KCNH1, Kv11.1) channels are expressed in heart, neurons, endocrine glands, and smooth muscle (reviewed in Perrin et al., 2008; Cheng and Claydon, 2012). Although initially identified by screening a human hippocampal cDNA library, HERG is actually important in regulating the heartbeat – in particular, the repolarization of the cardiac action potential as well as pacemaking behavior of the nodes of the heart (Piper et al., 2005). Mutations in this gene cause chromosome 7-associated long QT syndrome, a condition which predisposes patients to cardiac arrhythmias (Curran et al., 1995). Unlike most Kv channels, HERG are inward rectifiers, which function to limit the outflow of potassium ions during an action potential. However, compared to other inward rectifiers which block potassium conductance via an intracellular polyamine block, HERG channels prevent potassium outflow by rapid inactivation (Schonherr and Heinemann, 1996; Smith et al., 1996; Spector et al., 1996; Wang et al., 1997). Another distinguishing feature is that these channels activate and deactivate slowly compared to other Kv channels and that the inactivation process is voltage-dependent (Spector et al., 1996; Wang et al., 1997). They are closely related to the HCN channels, but despite the similarities, fundamental differences exist in their coupling mechanism; first, in the role of the CNBD, and second in the inactivation mechanism.

Previous evidence from Sanguinetti and Xu (1999) has shown that movement of the voltage sensor is coupled to channel activation via interaction of the S4–S5 linker with S6_T_. As in the HCN channels, this interaction seems to be mainly electrostatic; a single charge-reversal mutation of a residue on the S4–S5 linker, D540K, changed the channel’s properties, allowing reopening by hyperpolarization. Tristani-Firouzi et al. (2002) showed that this mutant phenotype could be reversed by introducing a complementary mutation at one specific amino acid, R665D, located at the S6 cytosolic end, which suggested that a D540K – R665 interaction is required for hyperpolarization-induced channel activation and that an electrostatic repulsion between the lysine and arginine is likely to underlie this altered channel behavior. This premise was further supported in the mutants D540R and D540K – R665K, which like D540K were also activated at hyperpolarizing potentials and have basic residues at both positions that exert a repulsive force. Simple neutralization of D540 (D540A), however, did not seem to be sufficient to efficiently uncouple the channel but still had significant influence on gating kinetics (Ng et al., 2012).

These results alone, however, could not clarify whether in the wildtype HERG channel the D540 and R665 residues form an electrostatic interaction that stabilizes the closed state at hyperpolarized potentials (Tristani-Firouzi et al., 2002). Interestingly, the addition of either I662A or L666A into the D540K mutant reduced the hyperpolarization induced inward current and increased the rate of deactivation, indicating that these two residues may modify the interaction between D540 and R665. Later work confirmed the involvement of these residues in coupling the voltage sensor movement with channel activation via the S4–S5 linker since a disulfide bond formation locked the channel in the closed state (Ferrer et al., 2006).

More recent work, alternatively, proposes that hydrophobic interactions may play a role in the electromechanical coupling between the S4–S5 linker and the S6 in HERG channels (Wynia-Smith et al., 2008). Based on homology modeling, V659 was found nestled within a hydrophobic pocket formed by S6, S5, and the S4–S5 linker residues in the closed state. Various mutations in this position disrupted channel closing, suggesting that these hydrophobic residues may be implicated in coupling pore and voltage sensor activation (Wynia-Smith et al., 2008). Residues C-terminal to V659 are more likely involved in S6–S6 interactions. In particular mutating the residues Q664, Y667, and S668 led to a constitutive leak current suggesting that they are involved either in closed-state stabilization or directly form the occluding gate at the bundle crossing (Wynia-Smith et al., 2008).

The importance of the S4–S5 linker is also underlined by the recent finding that other residues therein (S543, Y545, G546, and A548) variably influence activation/deactivation kinetics and steady state activation although the mechanism is not as well understood (Wang et al., 1998; Van Slyke et al., 2010; Ng et al., 2012). It seems clear that the S4–S5 linker is responsible for the slow kinetics of the HERG channel (Van Slyke et al., 2010). Thus far, coupling between S4–S5 linker and S6_T_ is conserved in HERG channels.

In contrast to HCN channel inactivation, however, HERG inactivation is not mediated by a loose coupling of S4–S5 linker and S6_T_ but by conformational changes at the outer pore region (Smith et al., 1996; Spector et al., 1996; Vaid et al., 2008; Kopfer et al., 2012). The process is thus similar to C-type inactivation observed in other Kv channels. In HERG channels, independent processes for C-type inactivation and pore opening are required because they open during deactivation but remain closed at hyperpolarized potentials. In addition, HERG channels, although containing a CNBD^5^ near the C-terminus (Warmke and Ganetzky, 1994), are not sensitive to cyclic nucleotides (Sanguinetti et al., 1995), which might be due to the low affinity (>51 μM) of cAMP to the binding site (Brelidze et al., 2009); however, even at high concentrations (10 mM) no effect on the current was observed. Muskett et al. (2011) proposed that instead the N-terminus, known to influence deactivation times, keeps the CNBD in the activated position, thereby stabilizing the open pore. The underlying reason might also be a lack in coupling between the CNBD and the coupling region (S4–S5 linker/S6T).

The CNBD seems to play a role mainly for assembly and trafficking of HERG (Akhavan et al., 2005). Thus in spite of the close relation to HCN channels, the electromechanical coupling mechanism of HERG resembles rather that of other Kv channels (Figure 4A).

### Large conductance calcium-activated potassium channels (BK_ca_)

Calcium and voltage-activated potassium channels (K_Ca_) are categorized into three major groups[large (BK), intermediate (IK), and small (SK) conductances] all of which are activated by both membrane depolarization and increases in intracellular calcium. K_Ca_ channels are comprised of α-subunit tetramers which assemble with auxiliary β-subunits that function to regulate sensitivity to calcium. In this review, we will concentrate on the BK_Ca_ channels, as their structure-function relations have been most intensively studied. BK channels are similar to Kv channels in that they contain voltage sensing (S1–S4) and pore (S5–S6) transmembrane regions but also differ because of the presence of additional domains: (i) a transmembrane helix S0, which interacts with the β-subunit (Wallner et al., 1996), (ii) a cytosolic domain made up of two regulator of potassium conductance (RCK) domains (RCK1 and RCK2) that contain high affinity calcium binding sites (Jiang et al., 2001), and finally (iii) a magnesium binding site, located at the interface between the cytosolic face of the voltage sensing domain and RCK1 (reviewed in Yang et al., 2008; Latorre et al., 2010; and Lee and Cui, 2010).

Although the gating mechanism is similar to other Kv channels (Diaz et al., 1998; Cui and Aldrich, 2000; Ma et al., 2006; Savalli et al., 2006), differences to the delayed rectifier Kv channels have been reported (Li and Aldrich, 2004, 2006; Zhou et al., 2011). The positive S4 residues do not as dominantly control the voltage dependence; instead charged residues throughout the voltage sensor domain lead to more global conformational changes (Ma et al., 2006; Savalli et al., 2006; Pantazis et al., 2009, 2010). Also the S0 segment has been suggested to be a functional part of the voltage sensor (Koval et al., 2007). Nevertheless, although it has not yet been shown directly, the structural similarities to the other Kv channels suggest that the coupling to the pore domain is mediated by the S4–S5 linker as in the other voltage-gated channels. Calcium dependence is modulated by mutations in the S4–S5 linker (Sullivan et al., 1997). In contrast, it has been shown that the intracellular RCK domain coordinates a magnesium ion not with the S4–S5 but the S0-S1 linker (Yang et al., 2008).

The RCK domains are responsible for modulating the voltage-dependent opening of BK_Ca_ by intracellular calcium (Jiang et al., 2001). Each monomer contains both RCK domains – specifically, a top ring consisting only of RCK1 and a bottom ring only consisting of RCK2 are formed (Yuan et al., 2010). Binding of Ca^2+^ to the RCK “gating ring” triggers a conformational change mainly in RCK1. The N-terminal lobe moves away from the central axis increasing the diameter by 12 Å. The N-terminus of the RCK domain is linked to the S6 via a 17 amino acid linker, leaving room to the possibility that the increased diameter of the gating ring pulls on the S6 decreasing the energy for the voltage sensor to open the pore. Implication of the S4–S5 linker in calcium dependent gating has been suggested early on based on mutagenesis data (Sullivan et al., 1997). Nevertheless, the interaction with the S0–S1 linker may also play a role (Yang et al., 2007, 2008). Coupling to voltage sensor and ligand binding site thus seems to be conserved also in BK_Ca_ channels. The distinct influence of the S0–S1 linker (which does not exist in other Kv channels) might reflect the more pronounced influence of the entire voltage sensor domain in BK_Ca_ channels.

A similar mechanism for channel opening by the RCK domains due to calcium binding has been proposed for opening of small calcium-activated potassium channels (SK_Ca_), where Ca/calmodulin binds to the Cam-binding domain (Schumacher et al., 2001). This chemomechanical coupling (Figure 4D) is similar to CNG channels. The difference between electro- and chemomechanical coupling is the link of the activator to the S4–S5 linker and the S6_T_, respectively.

## Concluding Remarks

We reviewed the mechanism of electromechanical coupling in various potassium channels, and found that the transfer of energy via annealing of the S4–S5 linker to the S6_T_ seems to be universally conserved throughout voltage-gated potassium channels (Figure 4). The manner of interaction (hydrophobic versus electrostatic) and the tightness of the coupling varies. “Decoupling” upon depolarization leads to hyperpolarization-activated channels (HCN) or to closed-state inactivation (Kv4, NavAB) according to whether the channel is open or closed at hyperpolarized potentials, respectively (Figures 4B,C). Another factor influencing the coupling is the pore’s default state. In channels where decoupling is required for channel closing, the pore has to close by default whereas channels which do not have an external energy source, such as the chemically activated KcsA channels, require the pore to open by default.

Annealing between the S4–S5 linker and the S6_T_ as part of electromechanical coupling also seems to be conserved among other voltage-gated ion channels and has been suggested for skeletal sodium channels (Muroi et al., 2010), a prokaryotic sodium channel (Payandeh et al., 2011, 2012; Yarov-Yarovoy et al., 2012), and voltage-gated calcium channels (Wall-Lacelle et al., 2011). It does not seem to be restricted to voltage-gated channels; some ligand-gated channels seem to follow a similar mechanism as has been shown, for instance, for the proton-gated KcsA channel (Thompson et al., 2008; Cuello et al., 2010a). Chemomechanical coupling involving a cytosolic ligand binding domain, however, is directly linked to the C-terminus of S6 (Schumacher et al., 2001; Zagotta et al., 2003; Taraska and Zagotta, 2010).

A number of familial diseases have been assigned to mutations located in the regions identified for electromechanical coupling including episodic ataxia (Rajakulendran et al., 2007), epilepsy (Escayg et al., 2000), long QT syndrome (Sanguinetti, 2010), and congenital deafness (Baig et al., 2011). Mutations in this region often do not eradicate channel function but rather modulate its voltage dependence, which might underlie the etiology of these non-fatal diseases. In this review, we saw that differences in the region of electromechanical coupling tune the channel, rendering it constitutively open, creating leaky channels or even reversing their voltage-dependence.

## Conflict of Interest Statement

The authors declare that the research was conducted in the absence of any commercial or financial relationships that could be construed as a potential conflict of interest.

## Footnotes

^1^Throughout the manuscript, “lower” and “upper” refer to the cytosolic and extracellular half of the transmembrane domains.

^2^Alternatively, this opening could be an intrinsic property of the selectivity filter, as observed in KcsA Cordero-Morales et al. (2006a).

^3^If the probability for pore opening was negligible, the cooperative models would become identical to a three-state sequential model. In this case, the state with open pore and resting voltage sensor would never be occupied.

^4^Numbering according to human HCN2 Acc. number EDL31671.

^5^Also called cyclic nucleotide binding homology domain, CNBHD, in HERG channels.
